# 2,2′,6,6′-Tetra­ethyl-4,4′-methyl­enedibenzonitrile

**DOI:** 10.1107/S1600536810020052

**Published:** 2010-06-05

**Authors:** Jingjing Yuan, Yanping Zhu

**Affiliations:** a Key Laboratory of Pesticides and Chemical Biology of the Ministry of Education, College of Chemistry, Central China Normal University, Wuhan 430079, People’s Republic of China

## Abstract

The asymmetric unit of the title compound, C_23_H_26_N_2_, contains one half-mol­ecule, which is completed by the operation of a crystallographic twofold axis. In the mol­ecule, the two benzene rings form a dihedral angle of 77.09 (7)°.

## Related literature

For applications of aromatic nitriles, see: Debasree *et al.* (2009 ▶); Lal Dhar *et al.* (2009 ▶); Ren *et al.* (2009 ▶); Zhou *et al.* (2009 ▶). For the preparation of the title compound, see: Donald *et al.* (1955 ▶).
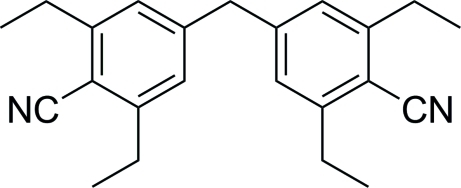


## Experimental

### 

#### Crystal data

C_23_H_26_N_2_
*M* *_r_* = 330.46
Monoclinic, 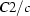

*a* = 16.016 (3) Å
*b* = 9.3218 (19) Å
*c* = 13.977 (3) Å
β = 115.55 (3)°
*V* = 1882.6 (7) Å^3^
*Z* = 4
Mo *K*α radiation
μ = 0.07 mm^−1^
*T* = 290 K
0.16 × 0.10 × 0.10 mm

#### Data collection

Bruker SMART 4K CCD area-detector diffractometer
8636 measured reflections
2055 independent reflections
1405 reflections with *I* > 2σ(*I*)
*R*_int_ = 0.028

#### Refinement

*R*[*F* ^2^ > 2σ(*F* ^2^)] = 0.038
*wR*(*F* ^2^) = 0.111
*S* = 1.16
2055 reflections
117 parameters
H-atom parameters constrained
Δρ_max_ = 0.24 e Å^−3^
Δρ_min_ = −0.20 e Å^−3^

### 

Data collection: *SMART* (Bruker, 1997 ▶); cell refinement: *SAINT* (Bruker, 1999 ▶); data reduction: *SAINT*; program(s) used to solve structure: *SHELXS97* (Sheldrick, 2008 ▶); program(s) used to refine structure: *SHELXL97* (Sheldrick, 2008 ▶); molecular graphics: *SHELXTL* (Sheldrick, 2008 ▶); software used to prepare material for publication: *SHELXTL*.

## Supplementary Material

Crystal structure: contains datablocks I, global. DOI: 10.1107/S1600536810020052/cv2718sup1.cif

Structure factors: contains datablocks I. DOI: 10.1107/S1600536810020052/cv2718Isup2.hkl

Additional supplementary materials:  crystallographic information; 3D view; checkCIF report

## Figures and Tables

**Fig. 1.**
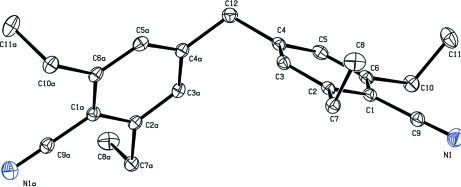
A view of (I), showing the atom-labelling scheme and 40% probability displacement ellipsoids [symmetry code: (a) -x, y, 1/2-z]. H atoms omitted for clarity.

## supplementary crystallographic information

### Comment

Aromatic nitriles are important intermediates in the synthesis of pharmaceuticals, agrochemicals, herbicides, dyes and pigments, and serve as  precursors for many useful compounds including benzoic acid derivatives, benzylamines, benzaldehydes, and heterocycles (Debasree *et al.*, 2009; Lal Dhar *et al.*, 2009; Ren *et al.*, 2009; Zhou *et al.*, 2009).

In this paper, we report the synthesis and crystal structure of the title compound (Fig. 1). In the molecule,  two benzene rings form a dihedral angle of 77.09 (7)°, and N···N separation is 11.67 (3)Å. The crystal packing doesn't exhibit hydrogen bonds or classical interactions.

### Experimental

The title compound has been synthesized following the known procedure (Donald *et al.*, 1955). To an ice-bath cooled solution of 4,4'-methylenebis(2,6-diethylaniline) and sodium nitrite in water was added dropwise concentrated hydrogen chloride, keeping the temperature at 0-5^o^C for 30 minutes. Then added potassium iodide into the mixed solution, and the white solid bis(3,5-diethyl-4-iodophenyl)methane was obtained. It reacted with cyanocopper in DMF solution at 180^o^C for 1 hour, then the title compound was obtained. X-ray quality crystal of the title compound was obtained by slow evaporation from chloroform solution at room temperature.

### Crystal data

**Table:**

C_23_H_26_N_2_	*F*(000) = 712
*M**_r_* = 330.46	*D*_x_ = 1.166 Mg m^−^^3^
Monoclinic, *C*2/*c*	Mo *K*α radiation, λ = 0.71073 Å
Hall symbol: -C 2yc	Cell parameters from 2049 reflections
*a* = 16.016 (3) Å	θ = 2.2–23.2°
*b* = 9.3218 (19) Å	µ = 0.07 mm^−^^1^
*c* = 13.977 (3) Å	*T* = 290 K
β = 115.55 (3)°	Block, colourless
*V* = 1882.6 (7) Å^3^	0.16 × 0.10 × 0.10 mm
*Z* = 4	

### Data collection

**Table:**

Bruker SMART 4K CCD area-detector diffractometer	1405 reflections with *I* > 2σ(*I*)
Radiation source: fine-focus sealed tube	*R*_int_ = 0.028
graphite	θ_max_ = 27.0°, θ_min_ = 3.2°
phi and ω scans	*h* = −20→20
8636 measured reflections	*k* = −11→11
2055 independent reflections	*l* = −17→17

### Refinement

**Table:**

Refinement on *F*^2^	Secondary atom site location: difference Fourier map
Least-squares matrix: full	Hydrogen site location: inferred from neighbouring sites
*R*[*F*^2^ > 2σ(*F*^2^)] = 0.038	H-atom parameters constrained
*wR*(*F*^2^) = 0.111	*w* = 1/[σ^2^(*F*_o_^2^) + (0.0506*P*)^2^ + 0.6433*P*] where *P* = (*F*_o_^2^ + 2*F*_c_^2^)/3
*S* = 1.16	(Δ/σ)_max_ = 0.003
2055 reflections	Δρ_max_ = 0.24 e Å^−^^3^
117 parameters	Δρ_min_ = −0.20 e Å^−^^3^
0 restraints	Extinction correction: *SHELXL97* (Sheldrick, 2008), Fc^*^=kFc[1+0.001xFc^2^λ^3^/sin(2θ)]^-1/4^
Primary atom site location: structure-invariant direct methods	Extinction coefficient: 0.0066 (13)

### Special details

**Table:**

Geometry. All esds (except the esd in the dihedral angle between two l.s. planes) are estimated using the full covariance matrix. The cell esds are taken into account individually in the estimation of esds in distances, angles and torsion angles; correlations between esds in cell parameters are only used when they are defined by crystal symmetry. An approximate (isotropic) treatment of cell esds is used for estimating esds involving l.s. planes.
Refinement. Refinement of *F*^2^ against ALL reflections. The weighted *R*-factor wR and goodness of fit *S* are based on *F*^2^, conventional *R*-factors *R* are based on *F*, with *F* set to zero for negative *F*^2^. The threshold expression of *F*^2^ > σ(*F*^2^) is used only for calculating *R*-factors(gt) etc. and is not relevant to the choice of reflections for refinement. *R*-factors based on *F*^2^ are statistically about twice as large as those based on *F*, and *R*- factors based on ALL data will be even larger.

### Fractional atomic coordinates and isotropic or equivalent isotropic displacement parameters (Å^2^)

**Table:**

	*x*	*y*	*z*	*U*_iso_*/*U*_eq_	
C1	0.15377 (8)	0.06614 (13)	0.12294 (10)	0.0215 (3)	
C2	0.17923 (8)	0.06280 (13)	0.23286 (10)	0.0214 (3)	
C3	0.12805 (8)	0.14471 (13)	0.27126 (10)	0.0217 (3)	
H3	0.1444	0.1449	0.3437	0.026*	
C4	0.05275 (8)	0.22672 (14)	0.20424 (10)	0.0222 (3)	
C5	0.02799 (8)	0.22437 (14)	0.09566 (10)	0.0239 (3)	
H5	−0.0233	0.2768	0.0504	0.029*	
C6	0.07756 (8)	0.14604 (14)	0.05298 (10)	0.0227 (3)	
C7	0.26297 (9)	−0.01923 (15)	0.30817 (10)	0.0254 (3)	
H7A	0.2723	−0.1023	0.2721	0.030*	
H7B	0.2524	−0.0530	0.3677	0.030*	
C8	0.34907 (10)	0.07400 (17)	0.34864 (13)	0.0379 (4)	
H8A	0.3615	0.1031	0.2902	0.057*	
H8B	0.4008	0.0205	0.3984	0.057*	
H8C	0.3394	0.1574	0.3829	0.057*	
C9	0.20907 (9)	−0.01286 (15)	0.08264 (10)	0.0251 (3)	
C10	0.05135 (9)	0.15086 (15)	−0.06463 (10)	0.0277 (3)	
H10A	−0.0150	0.1648	−0.1028	0.033*	
H10B	0.0664	0.0595	−0.0865	0.033*	
C11	0.10057 (11)	0.26976 (19)	−0.09430 (12)	0.0392 (4)	
H11A	0.0849	0.3607	−0.0742	0.059*	
H11B	0.0817	0.2684	−0.1695	0.059*	
H11C	0.1662	0.2554	−0.0580	0.059*	
C12	0.0000	0.3165 (2)	0.2500	0.0265 (4)	
H12	0.0432	0.3778	0.3053	0.032*	
N1	0.25510 (9)	−0.07416 (14)	0.05233 (10)	0.0370 (3)	

### Atomic displacement parameters (Å^2^)

**Table:**

	*U*^11^	*U*^22^	*U*^33^	*U*^12^	*U*^13^	*U*^23^
C1	0.0215 (6)	0.0215 (7)	0.0221 (6)	−0.0050 (5)	0.0099 (5)	−0.0016 (5)
C2	0.0203 (6)	0.0210 (7)	0.0229 (6)	−0.0039 (5)	0.0094 (5)	0.0005 (5)
C3	0.0223 (6)	0.0245 (7)	0.0183 (6)	−0.0037 (5)	0.0088 (5)	0.0003 (5)
C4	0.0203 (6)	0.0215 (6)	0.0263 (7)	−0.0048 (5)	0.0113 (5)	−0.0001 (5)
C5	0.0199 (6)	0.0241 (7)	0.0246 (7)	−0.0025 (5)	0.0066 (5)	0.0033 (5)
C6	0.0226 (6)	0.0235 (7)	0.0203 (6)	−0.0075 (5)	0.0077 (5)	0.0002 (5)
C7	0.0275 (7)	0.0270 (7)	0.0212 (7)	0.0034 (6)	0.0101 (6)	0.0026 (5)
C8	0.0263 (7)	0.0374 (9)	0.0385 (9)	0.0013 (6)	0.0031 (6)	0.0020 (7)
C9	0.0283 (7)	0.0266 (7)	0.0201 (6)	−0.0042 (6)	0.0102 (6)	−0.0004 (5)
C10	0.0273 (7)	0.0327 (8)	0.0195 (7)	−0.0039 (6)	0.0066 (6)	0.0002 (6)
C11	0.0393 (8)	0.0505 (10)	0.0266 (8)	−0.0096 (7)	0.0132 (7)	0.0069 (7)
C12	0.0255 (9)	0.0243 (10)	0.0309 (10)	0.000	0.0132 (8)	0.000
N1	0.0443 (7)	0.0378 (7)	0.0333 (7)	0.0020 (6)	0.0209 (6)	−0.0010 (6)

### Geometric parameters (Å, °)

**Table:**

C1—C6	1.4040 (18)	C7—H7B	0.9700
C1—C2	1.4096 (18)	C8—H8A	0.9600
C1—C9	1.4402 (18)	C8—H8B	0.9600
C2—C3	1.3865 (18)	C8—H8C	0.9600
C2—C7	1.5079 (18)	C9—N1	1.1486 (17)
C3—C4	1.3935 (18)	C10—C11	1.5178 (19)
C3—H3	0.9300	C10—H10A	0.9700
C4—C5	1.3935 (18)	C10—H10B	0.9700
C4—C12	1.5129 (16)	C11—H11A	0.9600
C5—C6	1.3885 (19)	C11—H11B	0.9600
C5—H5	0.9300	C11—H11C	0.9600
C6—C10	1.5115 (18)	C12—C4^i^	1.5130 (16)
C7—C8	1.5179 (19)	C12—H12	0.9700
C7—H7A	0.9700		
			
C6—C1—C2	121.79 (11)	H7A—C7—H7B	108.0
C6—C1—C9	119.69 (11)	C7—C8—H8A	109.5
C2—C1—C9	118.51 (11)	C7—C8—H8B	109.5
C3—C2—C1	117.92 (11)	H8A—C8—H8B	109.5
C3—C2—C7	120.36 (11)	C7—C8—H8C	109.5
C1—C2—C7	121.61 (11)	H8A—C8—H8C	109.5
C2—C3—C4	121.78 (12)	H8B—C8—H8C	109.5
C2—C3—H3	119.1	N1—C9—C1	178.28 (14)
C4—C3—H3	119.1	C6—C10—C11	112.67 (11)
C5—C4—C3	118.74 (12)	C6—C10—H10A	109.1
C5—C4—C12	121.37 (11)	C11—C10—H10A	109.1
C3—C4—C12	119.89 (10)	C6—C10—H10B	109.1
C6—C5—C4	121.96 (12)	C11—C10—H10B	109.1
C6—C5—H5	119.0	H10A—C10—H10B	107.8
C4—C5—H5	119.0	C10—C11—H11A	109.5
C5—C6—C1	117.78 (11)	C10—C11—H11B	109.5
C5—C6—C10	120.63 (12)	H11A—C11—H11B	109.5
C1—C6—C10	121.57 (12)	C10—C11—H11C	109.5
C2—C7—C8	111.18 (11)	H11A—C11—H11C	109.5
C2—C7—H7A	109.4	H11B—C11—H11C	109.5
C8—C7—H7A	109.4	C4—C12—C4^i^	112.85 (15)
C2—C7—H7B	109.4	C4—C12—H12	109.0
C8—C7—H7B	109.4		
			
C6—C1—C2—C3	−1.79 (18)	C4—C5—C6—C10	−177.34 (11)
C9—C1—C2—C3	177.02 (11)	C2—C1—C6—C5	0.88 (18)
C6—C1—C2—C7	−177.97 (11)	C9—C1—C6—C5	−177.92 (11)
C9—C1—C2—C7	0.85 (18)	C2—C1—C6—C10	179.15 (11)
C1—C2—C3—C4	0.93 (18)	C9—C1—C6—C10	0.35 (18)
C7—C2—C3—C4	177.15 (11)	C3—C2—C7—C8	−86.55 (15)
C2—C3—C4—C5	0.80 (18)	C1—C2—C7—C8	89.53 (15)
C2—C3—C4—C12	−178.92 (11)	C5—C6—C10—C11	89.82 (15)
C3—C4—C5—C6	−1.77 (19)	C1—C6—C10—C11	−88.39 (15)
C12—C4—C5—C6	177.94 (12)	C5—C4—C12—C4^i^	112.50 (12)
C4—C5—C6—C1	0.94 (18)	C3—C4—C12—C4^i^	−67.79 (10)

Symmetry codes: (i) −*x*, *y*, −*z*+1/2.
